# Association between the second-stage duration of labor and perinatal outcomes in women with a prior cesarean delivery

**DOI:** 10.1186/s12884-022-04871-0

**Published:** 2022-07-05

**Authors:** Yulian Li, Lizi Zhang, Lijun Huang, Yingyu Liang, Jingsi Chen, Shilei Bi, Weinan Deng, Lin Lin, Xiaoyi Wang, Luwen Ren, Shanshan Zeng, Minshan Huang, Baoying Huang, Yijian Zhang, Sushan Xie, Lili Du, Dunjin Chen

**Affiliations:** 1 Department of Obstetrics and Gynecology, Guangdong Provincial Key Laboratory of Major Obstetric Diseases, Guangdong Engineering and Technology Research Center of Maternal-Fetal Medicine, Guangdong-Hong Kong-Macao Greater Bay Area Higher Education Joint Laboratory of Maternal-Fetal Medicine, The Third Affiliated Hospital of Guangzhou Medical University, Guangzhou, PR China; 2 The Third Affiliated Hospital of Guangzhou Medical University, 63 Duobao Road, Liwan District, Guangzhou, 510150 China; 3 The Third Affiliated Hospital of Guangzhou Medical University, The Medical Center for Critical Pregnant Women in Guangzhou, 63 Duobao Road, Liwan District, Guangzhou, 510150 China

**Keywords:** TOLAC, VBAC, The second stage of labor, Maternal outcome, Neonatal outcome

## Abstract

**Background:**

The cesarean delivery (CD) rate has been increasing globally. Trial of labor after cesarean delivery (TOLAC) has been used as a key method for the reduction of the CD rate. Little is known, however, about the association between the second-stage duration of TOLAC and adverse maternal and neonatal outcomes. This study evaluated the association between perinatal outcomes and the duration of second-stage labor in women undergoing TOLAC.

**Methods:**

A 10-year retrospective cohort study was performed at the Department of Obstetrics and Gynecology, Third Affiliated Hospital of Guangzhou Medical University, between January 2010 and January 2020. Women undergoing TOLAC who reached the second stage of labor were included in this study. Duration of the second stage of labor was examined as a categorical variable (group I: <0.5 h, group II: 0.5–2 h and group III: ≥2 h) and as a continuous variable to evaluate the association with adverse perinatal outcomes by using multivariable regression models and a Cox proportional hazards regression model adjusting for potential confounders.

**Results:**

Of the 1,174 women who met the inclusion criteria, the median (interquartile range) length of the second stage was 0.5 h (0.3–0.9 h). Among them, 1,143 (97.4%) delivered vaginally and 31 underwent an unplanned CD. As the second-stage duration increased, operative vaginal delivery (OVD), CD, and postpartum hemorrhage (PPH) rates increased. Women in group III had higher risks of OVD (aOR = 11.34; 95% CI [5.06–25.41]), CD (aOR = 4.22; 95% CI [1.32–13.43]), and PPH (aOR = 2.43; 95% CI [1.31–4.50]) compared with group I. Correspondingly, blood loss and the oxytocin used to treat PPH increased significantly, while the postpartum hemoglobin reduced significantly in group III compared with group I. The incidence of uterine rupture, uterine atony, cervical laceration, red blood cell transfusion, and intensive care unit admission were similar in all three groups. Neonatal outcomes were not affected by the second-stage duration.

**Conclusions:**

Women undergoing TOLAC with second-stage duration of ≥2 h have higher odds of OVD, unplanned intrapartum CD, and PPH.

**Supplementary Information:**

The online version contains supplementary material available at 10.1186/s12884-022-04871-0.

## Background

The high rate of cesarean delivery (CD) is a medical problem as well as a global health issue [1]. The number of repeat CDs has risen proportionally as a result of the increased CD rate globally. Repeat CD, however, is associated with risks of adverse outcomes, such as placenta accreta, bladder and bowel damage, and uterine rupture [2]. The National Institutes of Health has suggested that trial of labor after cesarean delivery (TOLAC) is a reasonable option for many women with a prior CD and has called on organizations to facilitate access to TOLAC [3]. TOLAC is an effective strategy used to reduce maternal morbidity and risk of complications in future pregnancies as well as the overall CD rate at the population level. Vaginal birth after cesarean (VBAC) has fewer complications than elective repeat CD, but a failed TOLAC is also associated with increased risk of adverse perinatal outcomes [4, 5].

The duration of the second stage plays a vital role in the process of labor. The effect of the second-stage duration on labor outcomes has been studied widely and deeply in women without a previous uterine scar [6–12]. There is a paucity of data, however, focusing on the associations between the second-stage duration of TOLAC and perinatal outcomes.

We designed this study to evaluate the association between the second-stage duration of TOLAC and the perinatal outcomes. We found that the risk for operative vaginal delivery (OVD), CD, and postpartum hemorrhage (PPH) varied based on the length of the second stage in women who underwent TOLAC.

## Methods

### Study design and subject selection

We designed a retrospective cohort study in the Department of Obstetrics and Gynecology, Third Affiliated Hospital of Guangzhou Medical University, Guangzhou Medical Centre for Critical Pregnant Women, Guangzhou, China, between January 2010 and January 2020. This study was approved by the Medical Ethics Committee of Guangzhou Medical University with Medical Research No. 2016 (0406) on April 6, 2016. We reviewed the medical records of all women who had attempted vaginal labor after a prior CD and who had reached the second stage of labor.

Inclusion criteria were women with one prior CD of the transverse incision of the lower segment of uterus, singleton pregnancy, live birth, gestational age ≥37 weeks, and cephalic presentation at admission. The exclusion criteria included multiple gestations, non-cephalic presentation, placenta previa, placenta accreta, history of prior vaginal deliveries, multiple prior cesarean deliveries, antepartum stillbirth, and known lethal congenital anomalies. The second-stage duration of labor is defined as the time interval between full cervical dilation and delivery. Women were placed into one of three groups according to the second-stage duration of labor: group I (<0.5 h, 534 patients), group II (0.5–2 h, 559 patients), and group III (≥2 h, 81 patients). A second-stage duration of 0.5 h or shorter has been used in prior studies to define the short second stage [13]. The division at 2 h was based on a conventional threshold for defining a prolonged second stage of labor in a nulliparous woman without epidural analgesia [14]. Maternal and neonatal morbidities were defined based on clinical diagnoses.

### Maternal and labor characteristics

Maternal and labor characteristics, such as age, level of education, body mass index (BMI), gestational weeks at delivery, labor intervention (induction or augmentation of labor), indications of induction, epidural anesthesia, medical conditions (prelabor rupture of membranes [PROM], hypertensive disorders of pregnancy [HDP], gestational diabetes), length of the first stage, newborn sex, and birthweight were reviewed. Augmentation refers to the stimulation of uterine contractions when spontaneous contractions failed to result in progressive cervical dilation or descent of the fetus. Induction of labor (IOL) is indicated in situations in which the outcomes for mother and child are better if the pregnancy is not further prolonged.

### Outcome measures

In this study, PPH was defined as estimated blood loss ≥500 mL after vaginal delivery or ≥1000 mL after CD within 24 h [15]. The blood loss was estimated mainly through gravimetric measurement—namely, weight of bloody materials after subtracting dry weight of the same materials. Blood loss was quantified through volumetric measurement by collecting blood in graduated measurement containers. Other maternal outcomes included mode of delivery (spontaneous vaginal delivery [SVD], OVD, and CD), blood loss at 24 h after delivery, red blood cell transfusion, degree of postpartum decrease in hemoglobin, uterine rupture, uterine atony, cervical laceration, and use of oxytocin to treat PPH. Neonatal outcomes included Apgar score ≤7 (1 min), neonatal intensive care unit (NICU) admission, neonatal asphyxia, assisted ventilation, and neonatal infection.

### Statistical analysis

All analyses were performed using EmpowerStats (http://www.empowerstats.com) and the statistical package R (3.2.3 version). The second-stage duration was analyzed both as a continuous and as a categorical variable. Data were presented as n (%) or median (interquartile range). We performed comparisons between groups using chi-square or Fisher’s exact test for the dichotomous measure and the Kruskal–Wallis test for continuous variables. We used multivariable regression models to assess the association between second-stage duration and perinatal outcomes after adjusting for maternal age, BMI, level of education, gestational weeks at delivery, PROM, hypertensive disorders of pregnancy, gestational diabetes, induction, epidural anesthesia, the length of the first stage of labor, and birthweight. Confounders were based on existing literature and clinical judgment. We examined the association between the second-stage duration and mode of delivery by survival analyses using the log-rank test to compare Kaplan–Meier curves of cumulative events. We assessed the nonlinear relationship between the second-stage duration and PPH using Cox proportional hazards regression model based on cubic splines with 5 knots [16]. The optimal number of knots was assessed by comparing the likelihood ratios of nested models with 3–6 knots [17]. In the Cox model, the time of recording the blood loss within 24 h after delivery was used as the time scale. *P*-values of less than 0.05 indicated statistical significance.

## Results

**Fig. 1:**
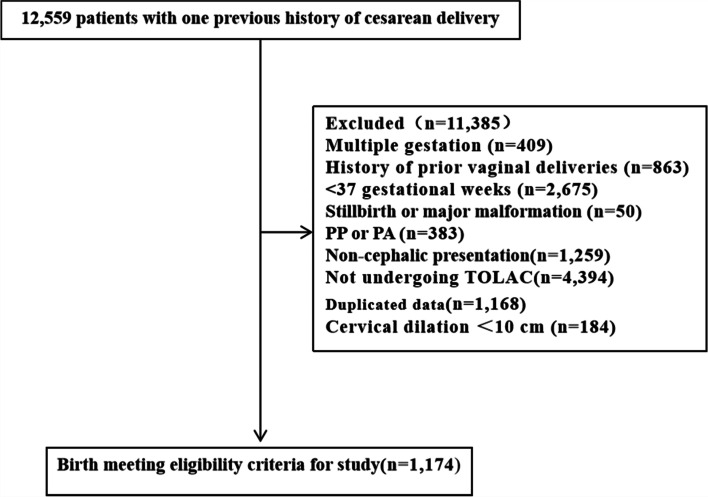
Flow diagram of cohort identification. TOLAC: trial of labor after cesarean delivery, PP: placenta previa, PA: placenta accreta

A total of 1,174 women met the inclusion criteria (Fig. 1); 534 patients delivered with the second-stage duration between 0 and 0.5 h, 559 between 0.5 and 2 h, and 81 ≥2 h.

Maternal and labor characteristics are presented in Table 1. Women in Group III had significantly advanced maternal age, longer first stage of labor, later gestational age, and higher birthweight than group I and group II. Higher rates of the second-stage duration ≥2 h were seen in women who had epidural anesthesia, gestational diabetes, IOL, and augmentation of labor. There were no differences in gravidity, maternal BMI, education level, PROM, hypertensive disorders of pregnancy, indications of induction, and fetal sex among groups (Table 1).

**Table 1:** Maternal and labor characteristics of women by length of second stage of labor

	Second stage length (h)			
Factors	<0.5 h (*n* = 534)	0.5–2.0 h (*n* = 559)	≥2.0 h (*n* = 81)	*P*-value
Gravidity	3.0 (2.0–3.0)	3.0 (2.0–3.0)	2.0 (2.0–3.0)	0.324
Age (y)				
≤25	43 (8.1%)	24 (4.3%)	2 (2.5%)	<0.001
25–30	154 (28.8%)	132 (23.6%)	11 (13.6%)	
30–35	221 (41.4%)	251 (44.9%)	38 (46.9%)	
≥35	116 (21.7%)	152 (27.2%)	30 (37.0%)	
Education				
High	290 (54.3%)	319 (57.1%)	52 (64.2%)	0.622
Middle	225 (42.1%)	219 (39.2%)	28 (34.6%)	
Low	8 (1.5%)	9 (1.6%)	1 (1.2%)	
Missing	11 (2.1%)	12 (2.1%)	0 (0.0%)	
BMI (kg/m^2^)				
≤25	146 (27.3%)	155 (27.7%)	25 (30.9%)	0.063
25–30	196 (36.7%)	227 (40.6%)	38 (46.9%)	
≥30	45 (8.4%)	59 (10.6%)	6 (7.4%)	
Missing	147 (27.5%)	118 (21.1%)	12 (14.8%)	
Gestational weeks at delivery (WK)				
37–38	52 (9.7%)	45 (8.1%)	1 (1.2%)	0.018
38–39	138 (25.8%)	135 (24.2%)	15 (18.5%)	
39–40	179 (33.5%)	220 (39.4%)	31 (38.3%)	
40–41	145 (27.2%)	127 (22.7%)	29 (35.8%)	
≥41	20 (3.7%)	32 (5.7%)	5 (6.2%)	
PROM				
No	440 (82.4%)	445 (79.6%)	60 (74.1%)	0.162
Yes	94 (17.6%)	114 (20.4%)	21 (25.9%)	
HDP				
No	527 (98.7%)	548 (98.0%)	81 (100.0%)	0.344
Yes	7 (1.3%)	11 (2.0%)	0 (0.0%)	
Gestational diabetes				
No	468 (87.6%)	467 (83.5%)	63 (77.8%)	0.028
Yes	66 (12.4%)	92 (16.5%)	18 (22.2%)	
Labor intervention				
Spontaneous of labor	341 (63.9%)	303 (54.2%)	15 (18.5%)	<0.001
Augmentation of labor	139 (26.0%)	212 (37.9%)	53 (65.4%)	
Induction of labor	54 (10.1%)	44 (7.9%)	13 (16.0%)	
Indications of induction				
Delayed pregnancy	32 (59.3%)	24 (54.5%)	8 (61.5%)	0.856
Gestational complications	22 (40.7%)	20 (45.5%)	5 (38.5%)	
Epidural anesthesia				
No	518 (97.0%)	518 (92.7%)	65 (80.2%)	<0.001
Yes	16 (3.0%)	41 (7.3%)	16 (19.8%)	
First stage length (h)	5.8 (3.8–9.0)	7.2 (5.0–11.2)	9.3 (6.4–14.3)	<0.001
Birthweight (g)				
<3,500	456 (85.4%)	418 (74.8%)	46 (56.8%)	<0.001
≥3,500	78 (14.6%)	141 (25.2%)	35 (43.2%)	
Fetal sex				
Boys	251 (47.0%)	274 (49.0%)	28 (34.6%)	0.052
Girls	283 (53.0%)	285 (51.0%)	53 (65.4%)	

*BMI* Body mass index, *PROM* Prelabor rupture of membranes, *HDP* Hypertensive disorders of pregnancy

Gestational complications include hypertensive disorders of pregnancy, gestational diabetes, oligohydramnios, and so on

Data are presented as n (%) or median (interquartile range)

**Fig. 2:**
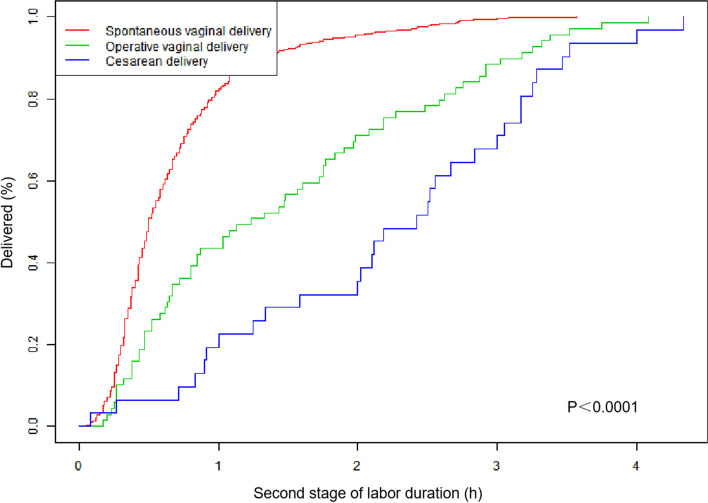
Kaplan–Meier curves of cumulative events examining the length of the second stage of labor and mode of delivery (*P* < 0.0001 by log-rank test)

Among the included women, 1,143 (97.4%) had a successful TOLAC. The rate of VBAC decreased, whereas OVDs and CDs increased with prolonged second-stage length. As shown in Table 2, the rate of VBAC was high in group I (98.5%) and group II (98.0%), but fell to 85.2% in group III, whereas CD rates increased from 1.5% in group I and 2.0% in group II to 14.8% in group III. For VBAC, the rate of OVD increased from 3.0% in group I to 5.9% in group II and 24.7% in group III (*P* < 0.001). Women with SVD had a shorter second stage compared with those with OVD or CD (*P*<0.001 by log-rank test; Fig. 2). Furthermore, women with a prolonged second stage of labor (≥2 h) were more likely to be undergoing OVD for the prolonged second stage as the indication. Of the women who had a CD, 45.2% were performed because labor failed to progress (Table 2). The association between the second-stage duration and operative delivery remained statistically significant when potential confounding factors were controlled in the multivariable regression models (Table 3).

**Table 2:** Maternal and neonatal outcomes by length of second stage of labor

Length of the second stage	<0.5 h (*n* = 534)	0.5–2.0 h (*n* = 559)	≥2.0 h (*n* = 81)	*P*-value
Mode of delivery				
Spontaneous vaginal (*n* = 1074)	510 (95.5%)	515 (92.1%)	49 (60.5%)	<0.001
Operative vaginal (*n* = 69)	16 (3.0%)	33 (5.9%)	20 (24.7%)	
Cesarean section (*n* = 31)	8 (1.5%)	11 (2.0%)	12 (14.8%)	
Indications of OVD				
Prolonged second stage labor (*n* = 15)	0 (0.0%)	0 (0.0%)	15 (75.0%)	<.0001
Nonreassuring fetal heart rate (*n* = 51)	16 (100.0)	31 (93.9%)	4 (20.0%)	
Failed progress in labor (*n* = 3)	0 (0.0%)	2 (6.1%)	1 (5.0%)	
Indications of cesarean				
Failed progress in labor (*n* = 14)	0 (0.0%)	6 (54.5%)	8 (66.7%)	0.048
Nonreassuring fetal heart rate (*n* = 14)	7 (87.5%)	4 (36.4%)	3 (25.0%)	
Others (*n* = 3)	1 (12.5%)	1 (9.1%)	1 (8.3%)	
Maternal outcomes				
PPH	60 (11.2%)	91 (16.3%)	22 (27.2%)	<0.001
Blood loss at 24 h (mL)	315.0 (284.0–390.0)	331.0 (290.0–453.5)	420.0 (310.0–564.0)	<0.001
Hb decreased (g/L)	16.0 (9.0–23.0)	18.0 (11.0–25.0)	22.0 (14.0–30.0)	<0.001
Oxytocin used (U)	20.0 (10.0–20.0)	20.0 (10.0–20.0)	20.0 (20.0–40.0)	<0.001
Transfusion	14 (2.6%)	18 (3.2%)	4 (4.9%)	0.508
Uterine atony	10 (1.9%)	13 (2.3%)	2 (2.5%)	0.853
Cervical laceration^a^	50 (9.5%)	42 (7.7%)	6 (8.7%)	0.559
Uterine rupture	7 (1.3%)	1 (0.2%)	1 (1.2%)	0.088
ICU	2 (0.4%)	0 (0.0%)	0 (0.0%)	0.301
Neonatal outcomes				
1-min Apgar score ≤7	7 (1.3%)	3 (0.5%)	2 (2.5%)	0.181
5-min Apgar score ≤7	2 (0.4%)	0 (0.0%)	0 (0.0%)	0.301
Neonatal asphyxia	6 (1.1%)	2 (0.4%)	1 (1.2%)	0.308
Assisted ventilation	7 (1.3%)	3 (0.5%)	2 (2.5%)	0.181
NICU	12 (2.2%)	20 (3.6%)	4 (4.9%)	0.265
Infection	8 (1.5%)	21 (3.8%)	3 (3.7%)	0.062

*PPH* Postpartum hemorrhage, *Hb* Hemoglobin, *ICU* Intensive care unit, *NICU* Neonatal intensive care unit

^a^Vaginal deliveries only

Data was presented as n (%) or median (interquartile range)

**Table 3:** Adjusted models of perinatal outcomes in multivariate regression analyses by length of the second stage of labor

	Nonadjusted model		Adjusted model^c^	
Length of the second stage^b^	0.5–2.0 h (*n* = 559)	≥2.0 h (*n* = 81)	0.5–2.0 h (*n* = 559)	≥2.0 h (*n* = 81)
Mode of delivery				
Operative vaginal	2.03 (1.10–3.74)	10.61 (5.22–21.57)	1.97 (1.05–3.69)	11.34 (5.06–25.41)
Cesarean section	1.32 (0.53–3.31)	11.43 (4.52–28.96)	0.63 (0.22–1.80)	4.22 (1.32–13.43)
Maternal outcomes				
PPH	1.54 (1.08–2.18)	2.95 (1.69–5.15)	1.39 (0.96–2.02)	2.43 (1.31–4.50)
Blood loss at 24 h (ml)^a^	32.54 (3.49–61.60)	116.68 (59.43–173.94)	22.47 (7.19–52.13)	86.89 (27.15–146.64)
Hb decreased (g/L)^a^	2.38 (0.99–3.78)	6.63 (3.89–9.37)	1.87 (0.43–3.31)	5.74 (2.85–8.64)
Oxytocin used (U)^a^	1.52 (−0.21–3.25)	12.29 (8.88–15.69)	0.21 (−1.47–1.89)	8.20 (4.82–11.59)
Red blood cell transfusion	1.24 (0.61–2.51)	1.93 (0.62–6.01)	1.04 (0.49–2.21)	1.32 (0.38–4.56)
Uterine rupture	0.13 (0.02–1.10)	0.94 (0.11–7.75)	0.14 (0.02–1.25)	0.95 (0.09–10.45)
Neonatal outcomes				
1-min Apgar score ≤ 7	0.41 (0.10–1.58)	1.91 (0.39–9.34)	0.38 (0.09–1.71)	1.36 (0.22–8.49)
Neonatal asphyxia	0.32 (0.06–1.57)	1.10 (0.13–9.26)	0.18 (0.03–1.10)	0.44 (0.03–7.56)
Infection	2.57 (1.13–5.85)	2.53 (0.66–9.74)	2.00 (0.84–4.75)	0.91 (0.21–4.04)
NICU	1.61 (0.78–3.34)	2.26 (0.71–7.18)	1.33 (0.62–2.83)	1.13 (0.32–4.04)

*PPH* Postpartum hemorrhage, *Hb* Hemoglobin, *NICU* Neonatal intensive care unit, *CI* Confidence interval

^a^Continuous variables are presented as β (95% CI); other variables are presented as OR (95% CI)

^b^Reference comparison group: Women with second stage of labor between 0 and 0.5 h

^c^Adjusting for maternal age, BMI, level of education, gestational weeks at delivery, PROM, HDP, GDM, induction, epidural anesthesia, and the length of first stage of labor and birthweight

**Fig. 3:**
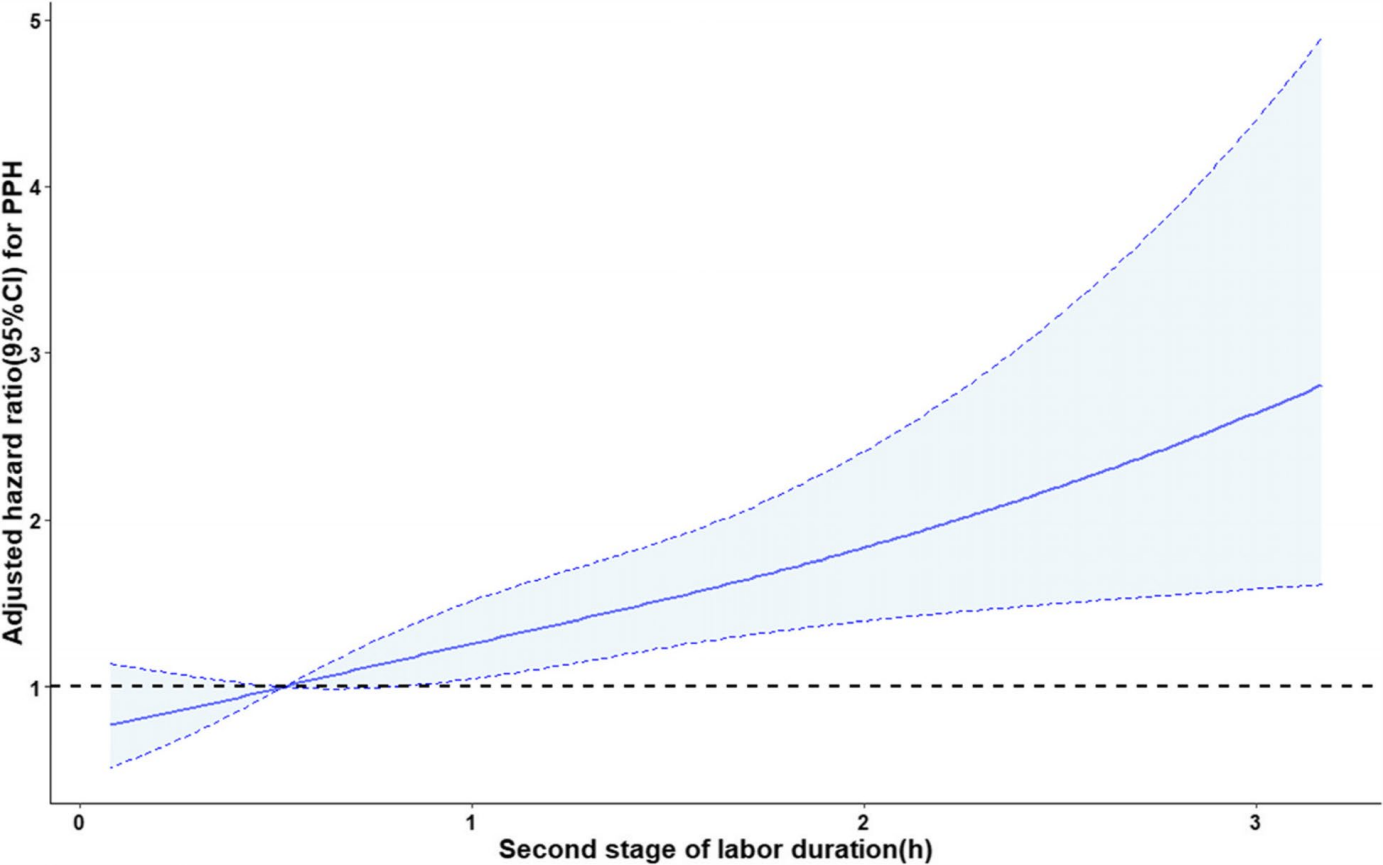
Second stage of labor and PPH risk. Adjusted hazard ratio and 95% pointwise confidence bands for association between duration of second stage of labor and PPH

Regarding maternal outcomes (Table 2), no differences were observed in uterine rupture, uterine atony, cervical laceration, red blood cell transfusion, and ICU admission as the second-stage duration increased. Incidences of PPH increased from 11.2% in group I and 16.3% in group II to 27.2% in group III (*P* < 0.001). When these associations were further examined by multivariable regression analysis controlling for potential confounding factors and using women with a second stage between 0 and 0.5 h as the reference group, women with second-stage duration ≥2 h had higher odds of PPH (aOR = 2.43; 95% CI [1.31–4.50]) (Table 3). Similarly, blood loss at 24 h after delivery (aβ = 86.89; 95% CI [27.15–146.64]) was also higher in group III. Correspondingly, the use of oxytocin to treat PPH increased significantly (aβ = 8.20; 95% CI [4.82–11.59]). Moreover, postpartum hemoglobin (aβ = 5.74; 95% CI [2.85–8.64]) reduced significantly in group III (Table 3). We also examined second-stage labor duration as a continuous variable, which was modeled using regression splines. The hazard of PPH trended higher with longer duration of second-stage labor (Fig. 3).

In comparison, neonatal outcomes (low Apgar scores, neonatal asphyxia, NICU admission, assisted ventilation, and neonatal infection) were not statistically different among the three groups, both for unadjusted and adjusted outcomes (Table 3).

We further analyzed the association between the length of the second stage and perinatal outcomes in women who delivered vaginally (see Table S1) and observed that the risk of operative interventions and PPH was increased as the duration of the second stage of labor takes longer, but neonatal outcomes were not affected by the duration of the second stage of labor.

## Discussion

In this study, we investigated the association between perinatal outcomes and the second-stage duration of labor in women who underwent TOLAC. We found that increased second-stage duration of labor was associated with a higher risk of OVD, CD, and PPH but not with other adverse maternal and neonatal outcomes.

The second stage is the most critical stage of labor. The effect of the duration of the second stage of labor on perinatal outcomes has been well studied. E. B. Ausbeck’s retrospective cohort study showed that prolonged second stage was associated with adverse maternal outcomes such as PPH, chorioamnionitis, operative complications, postpartum infections, and ICU admission, but not with adverse neonatal outcomes [18]. Several studies also reported that multiparous women were at increased risk for operative deliveries, peripartum morbidity, and adverse neonatal outcomes with the second stage of 3 h or greater [7, 10]. A randomized controlled study (RCT) that included 78 women, however, showed that women with a prolonged second stage of labor could increase the rate of vaginal delivery, and the maternal and neonatal adverse outcomes were not statistically significant [19]. Although no maternal morbidity differences were found in this RCT, PPH, endometritis, and third- and fourth-degree lacerations all had wide confidence intervals. Additionally, the time between the extended labor group and usual labor group (92 ± 65 min vs. 78 ± 46 min) was not statistically different. This RCT was underpowered to determine differences in these outcomes. Notably, the associations of prolonged second stage and adverse maternal and neonatal outcomes remain controversial. Moreover, women undergoing TOLAC were not included in these studies. Comparatively, little is known about the risk of a longer second-stage duration of labor for women undergoing TOLAC, and hence, a standard for the second stage specifically for TOLAC is lacking.

Our study showed that women with SVD had a shorter second stage than those with OVD or CD, which was in line with findings from other studies [20, 21]. These women were more likely not to have had any labor interventions (augmentation of labor or IOL). Furthermore, we found that women with a prolonged second stage of labor (≥2 h) were more likely to be undergoing OVD or CD for prolonged second stage or failed progress in labor as the indication. Thus, the risk factors associated with failed progress in labor and prolonged stage of labor should be identified and intervened in the second stage, such as uterine atony, fatigue, insufficient energy intake, and fetal malposition. Some harmless measures, such as free position or immediate pushing, should be taken to shorten the second-stage duration.

Regarding adverse perinatal outcomes, our study found no differences in uterine rupture, uterine atony, cervical laceration, red blood cell transfusion, and ICU admission as the second-stage duration increased. In contrast, Hehir’s prospective observational cohort study showed that the risk of adverse maternal outcomes (chorioamnionitis, endometritis, red cell transfusion, and uterine rupture or dehiscence) increased with the length of the second stage in women who underwent TOLAC. One possible explanation for these differences may be related to different study timelines and obstetric populations [22]. The Hehir’s study was conducted 20 years ago and medical resources and practice patterns have changed over the past few decades. The induction rate of our cohort was 9%, whereas Hehir’s study was 23% and included unadjusted analyses of adverse outcomes, which may explain the different results, as studies have shown that induced labor increases the risk of adverse outcomes [23, 24]. In addition, we found that PPH was related to the second-stage duration, even after adjusting confounding factors. PPH remains the leading cause of maternal deaths from direct obstetric causes, and therefore it is critical to obtain proper assessments of PPH. Considering that there were certain subjective biases in the measurement of postpartum blood loss in clinical work, we also evaluated variables related to blood loss, such as changes in postpartum hemoglobin and oxytocin used for the treatment of PPH, to increase the credibility of the results. According to our study results, with the increased incidence of PPH, the oxytocin used for PPH treatment also increased. Correspondingly, the postpartum hemoglobin decreased, indicating that the risk of PPH rises with an extended duration of the second stage of labor.

Moreover, women with a prior CD were previously reported to be less likely to attempt a subsequent vaginal delivery in China, presumably because of fear of adverse outcomes associated with TOLAC [25, 26]. The change of birth policies in China in recent years, however, from a one-child policy to a two-child policy to the current three-child policy, offers an opportunity to promote VBAC in China. Our study about the association between the second-stage duration of labor and perinatal outcomes in women undergoing TOLAC could provide clinical support for implementing VBAC. In addition, the rate of successful TOLAC in our study was relatively high (97.4%) as compared with Hehir’s study [22]. The main reason is that the current cohort primarily includes women who underwent spontaneous labor, and these women are more likely to have a successful VBAC [27]. The indications for TOLAC also may differ in different countries. Nevertheless, the current findings regarding the favorable VBAC rates among women with lower risk imply that TOLAC should not be discouraged in this setting.

This study provides information regarding the association between second-stage duration and mode of delivery as well as associated perinatal outcomes in women undergoing TOLAC; however, it also has some limitations. Retrospective studies are inherently limited by the data sets used. In addition, because this was a cohort study from a single center in China, differences in practice style or patient preferences may have been unique to the study cohort, and this study was likely underpowered to investigate significant clinical differences in severe obstetric outcomes, such as uterine rupture, postpartum hysterectomy, or rare neonatal morbidities. The small sample size also presented a limitation in our study. Multivariable models with larger populations may be better able to characterize the relationship between the second-stage length and adverse outcomes. Thus, caution is warranted when extending our findings to other populations.

## Conclusions

Our study revealed that the prolonged (≥2 h) second stage of labor may lead to increased OVD and CD rate, as well as the incidence of PPH in women who underwent TOLAC. Neonatal outcomes were similar by the second-stage length. The benefits of the prolonged second stage of labor to promote the rate of vaginal delivery should be weighed against the increased operative delivery and PPH rate. In our study, we observed that women with a shorter duration of second-stage labor and lower labor intervention rates were more likely to have a successful VBAC. When labor progresses slowly, identifying and treating risk factors associated with failed progress in labor and prolonged stage of labor in the second stage (such as uterine atony, fetal malposition) might increase the chance for a successful VBAC. Our data could aid in counseling and management of the second stage of TOLAC.

## Supplementary Information

**Additional file 1: Table S1.** Adjusted models of perinatal outcomes in multivariate regression analyses in successful VBAC by length of the second stage of labor. Analyzed the association between the length of the second stage and perinatal outcomes in women who delivered vaginally.

## Data Availability

The datasets generated and/or analyzed during the current study are not publicly available due to the confidentiality of patient information but are available from the corresponding author on reasonable request.

## Abbreviations

TOLAC: Trial of labor after cesarean delivery
CD: Cesarean delivery
OVD: Operative vaginal delivery
aOR: Adjusted odds ratio
CI: Confidence interval
PP: Placenta previa
PA: Placenta accrete
BMI: Body mass index
PROM: Prelabor rupture of membranes
AROM: Artificial rupture of membranes;
VBAC: Vaginal birth after cesarean
PPH: Postpartum hemorrhage
Hb: Hemoglobin
ICU: Intensive care unit
NICU: Neonatal intensive care unit
