# Supplementary material for: Association between the second-stage duration of labor and perinatal outcomes in women with a prior cesarean delivery
Source: BMC Pregnancy Childbirth. 2022 Jul 5;22:543. doi: 10.1186/s12884-022-04871-0 (PMC9254554; doi:10.1186/s12884-022-04871-0)
Supplement: Supplementary file 1 — Additional file 1: Table S1. Adjusted models of perinatal outcomes in multivariate regression analyses in successful VBAC by length of the second stage of labor. Analyzed the association between the length of the second stage and perinatal outcomes in women who delivered vaginally. [file 12884_2022_4871_MOESM1_ESM.doc]

**Supplemental table**

S1 table. Adjusted models of perinatal outcomes in multivariate regression analyses in successful VBAC by length of the second stage of labor. Analyzed the association between the length of the second stage and perinatal outcomes in women who delivered vaginally. (doc)

| **S1 table. Adjusted models of perinatal outcomes in multivariate regression analyses in successful VBAC by length of the second stage of labor.** | | | | | |
| --- | --- | --- | --- | --- | --- |
|  | Non-adjusted model | |  | Adjusted model^⋕^ | |
| Length of the second stage^$^ | 0.5–2.0 h (n = 548) | ≥2 h (n = 69) |  | 0.5–2.0 h (n = 548) | ≥2 h (n = 69) |
| Operative vaginal | 2.04 (1.11–3.76) | 13.01 (6.33–26.72) |  | 1.94 (1.03–3.65) | 12.51 (5.55–28.17) |
| Maternal outcomes |  |  |  |  |  |
| PPH | 1.55 (1.09–2.20) | 3.64 (2.05–6.45) |  | 1.86 (0.44–3.28) | 3.40 (1.84–6.27) |
| Blood loss at 24 h (ml)* | 32.20 (2.65–61.75) | 120.22 (58.23–182.20) |  | 21.59 (-8.66–51.84) | 93.24 (29.36–157.12) |
| Hb decreased (g/L)* | 2.21 (0.80–3.61) | 7.33 (4.40–10.26) |  | 0.56 (0.11–1.01) | 1.47 (0.51–2.43) |
| Oxytocin used (U)* | 1.23 (-0.37–2.83) | 7.32 (3.97–10.67) |  | 0.54 (-1.05–2.13) | 5.50 (2.13–8.86) |
| Transfusion | 1.26 (0.61–2.63) | 2.43 (0.77–7.67) |  | 1.06 (0.48–2.32) | 1.70 (0.49–5.91) |
| Uterine rupture | 0.16 (0.02–1.32) | 1.27 (0.15–10.75) |  | 0.18 (0.02–1.64) | 1.16 (0.10–13.01) |
| Cervical laceration | 0.79 (0.51–1.21) | 0.91 (0.37–2.20) |  | 0.81 (0.52–1.28) | 0.90 (0.35–2.31) |
| Neonatal outcomes |  |  |  |  |  |
| 1-min Apgar score ≤ 7 | 0.57 (0.14–2.41) | 1.53 (0.18–13.31) |  | 0.91 (0.17–4.80) | 1.73 (0.14–21.00) |
| Neonatal asphyxia | 0.38 (0.07–1.98) | - |  | 0.33 (0.05–2.40) | - |
| Infection | 2.33 (1.01–5.36) | 0.95 (0.12–7.73) |  | 1.92 (0.80–4.61) | 0.41 (0.04–3.72) |
| NICU | 1.68 (0.79–3.57) | 1.40 (0.30–6.44) |  | 1.40 (0.64–3.07) | 0.78 (0.16–3.89) |
| VBAC: Vaginal birth after cesarean, PPH: postpartum hemorrhage, Hb: hemoglobin, NICU: neonatal intensive care unit; CI: confidence interval;  *Continuous variables are presented as β (95% CI), other variables are presented as OR (95% CI);  ^$^Reference comparison group: Women with second stage of labor between 0 and 0.5 h; ^⋕^Adjusting for maternal age, BMI, level of education, gestational weeks at delivery, PROM, HDP, GDM, induction, epidural anesthesia, the length of first stage of labor and birthweight. | | | | | |
